# Supplementary material for: Development and calibration of a mathematical model of HIV outcomes among Rwandan adults: Informing achievement of global targets across sub-populations in Rwanda
Source: PLoS One. 2025 May 14;20(5):e0310662. doi: 10.1371/journal.pone.0310662 (PMC12077668; doi:10.1371/journal.pone.0310662)
Supplement: S1 File — (PDF) [file pone.0310662.s001.pdf]

Development and calibration of a mathematical model of HIV outcomes among Rwandan adults: informing achievement of global targets across sub-populations in Rwanda

Supplementary Content

April D. Kimmel, Zhongzhe Pan, Ellen Brazier, Gad Murenzi, Deo Mujwara, Benjamin Muhoza, Marcel Yotebieng, Kathryn Anastos, Denis Nash

Contents

List of Figures……………………………………………………………………………..3
List of Tables 3
Supplemental Methods.. 4
1 System of equations 4
2 Force of infection 7
3 Parameterizing the force of infection equation 9
4 Model initialization 13
5 Transition probabilities 17
5.1 HIV diagnosis 17
5.2 Natural history 19
5.3 Linkage to care.. 19
5.4 Lost to follow-up.. 20
5.5 Death 22
6 Calibration targets 23
Supplemental Results.. 25
References... 28

List of Figures

Figure S1. Sub-populations and mixing patterns in the CA-IeDEA model.................................. 8

List of Tables

Table S1. Model compartments: a legend for the system of equations
Table S2. CA-IeDEA HIV policy model parameters
Table S3. Probabilities and multipliers for the force of infection, by sub-population and direction
Table S4. Proportion of consistent condom use, by sub-population
Table S5. Initial population size, by sub-population and HIV status
Table S6. Parameters to initialize the model
Table S7. Average monthly growth rate for sub-populations*
Table S8. Monthly probability of HIV diagnosis, by sub-population and CD4 stratum
Table S9. Monthly probabilities of natural history disease progression
Table S10. Monthly probability of sub-population linkage to HIV care, by CD4 stratum
Table S11. Monthly probabilities of LTFU, on ART and suppressed, and viral failure
Table S13. Annual probability of sub-population death, by CD4 stratum and care engagement*
Table S14. HIV epidemic targets, by sub-population
Table S15. HIV care continuum targets, 15–49 years
Table S16. Number of people on ART, 15–49 years

Supplemental Methods

The model is a dynamic epidemiologic transmission model, simulating HIV disease spread over time. The model is an extended SIR model, where *S* is susceptible, *I* is infectious, and *R* is removed (i.e. death). The infectious compartment is further adapted so that it contains 26 compartments, which are defined by disease progression and steps along the HIV care continuum. The model captures 35 different sub-populations defined by sex, age group, risk of HIV acquisition (female sex workers vs. others, men who have sex with men (MSM) vs others) and urbanicity. Each of the model's 35 sub-populations is represented by the compartments described, resulting in a total of 910 model compartments (35 sub-populations x 26 compartments = 910 sub-population compartments).

1 System of equations

Epidemic dynamics and disease progression are captured by a system of equations. While systems of equations in dynamic models are typically expressed using ordinary differential equations, we implement the system using a discrete approximate of the derivatives in a system of difference equations, similar to other modeling studies [1]. The system of equations is applied to each of the 35 sub-populations. The system of difference equations is shown below. Model compartments and parameters in the system of equations are described in Error! Reference source not found. and Error! Reference source not found..

$S_t = S_{t-1} + \rho_t S_{t-1} - \lambda_{tr} S_{t-1}$
$X_{1,t} = X_{1,t-1} + \lambda_{tr} S_{t-1} - \delta_1 X_{1,t-1} - \alpha_1 X_{1,t-1} - \mu_1 X_{1,t-1}$
$X_{2,t} = X_{2,t-1} + \delta_1 X_{1,t-1} - \delta_2 X_{2,t-1} - \alpha_2 X_{2,t-1} - \mu_2 X_{2,t-1}$
$X_{3,t} = X_{3,t-1} + \delta_2 X_{2,t-1} - \delta_3 X_{3,t-1} - \alpha_3 X_{3,t-1} - \mu_3 X_{3,t-1}$
$X_{4,t} = X_{4,t-1} + \delta_3 X_{3,t-1} - \alpha_4 X_{4,t-1} - \mu_4 X_{4,t-1}$
$X_{5,t} = X_{5,t-1} + \alpha_1 X_{1,t-1} - \delta_1 X_{5,t-1} - \sigma_1 X_{5,t-1} - \mu_1 X_{5,t-1}$
$X_{6,t} = X_{6,t-1} + \alpha_2 X_{2,t-1} + \delta_1 X_{5,t-1} - \delta_2 X_{6,t-1} - \sigma_2 X_{6,t-1} - \mu_2 X_{6,t-1}$
$X_{7,t} = X_{7,t-1} + \alpha_3 X_{3,t-1} + \delta_2 X_{6,t-1} - \delta_3 X_{7,t-1} - \sigma_3 X_{7,t-1} - \mu_3 X_{7,t-1}$
$X_{8,t} = X_{8,t-1} + \alpha_4 X_{4,t-1} + \delta_3 X_{7,t-1} - \sigma_4 X_{8,t-1} - \mu_4 X_{8,t-1}$
$X_{9,t} = X_{9,t-1} + \sigma_1 X_{5,t-1} - \delta_4 X_{9,t-1} - \Theta_1 X_{9,t-1} - \gamma_1 X_{9,t-1} - \mu_5 X_{9,t-1}$
$X_{10,t} = X_{10,t-1} + \sigma_2 X_{6,t-1} + \delta_4 X_{9,t-1} - \delta_5 X_{10,t-1} - \Theta_2 X_{10,t-1} - \gamma_2 X_{10,t-1} - \mu_6 X_{10,t-1}$
$X_{11,t} = X_{11,t-1} + \sigma_3 X_{7,t-1} + \delta_5 X_{10,t-1} - \delta_6 X_{11,t-1} - \Theta_3 X_{11,t-1} - \gamma_3 X_{11,t-1} - \mu_7 X_{11,t-1}$
$X_{12,t} = X_{12,t-1} + \sigma_4 X_{8,t-1} + \delta_6 X_{11,t-1} - \Theta_4 X_{12,t-1} - \gamma_4 X_{12,t-1} - \mu_8 X_{12,t-1}$
$X_{13,t} = X_{13,t-1} + \gamma_1 X_{9,t-1} + \gamma_5 X_{17,t-1} + \gamma_9 X_{21,t-1} - \delta_1 X_{13,t-1} - \mu_1 X_{13,t-1}$
$X_{14,t} = X_{14,t-1} + \gamma_2 X_{10,t-1} + \gamma_6 X_{18,t-1} + \gamma_{10} X_{22,t-1} + \delta_1 X_{13,t-1} - \delta_2 X_{14,t-1} - \mu_2 X_{14,t-1}$
$X_{15,t} = X_{15,t-1} + \gamma_3 X_{11,t-1} + \gamma_7 X_{19,t-1} + \gamma_{11} X_{23,t-1} + \delta_2 X_{14,t-1} - \delta_3 X_{15,t-1} - \mu_3 X_{15,t-1}$
$X_{16,t} = X_{16,t-1} + \gamma_4 X_{12,t-1} + \gamma_8 X_{20,t-1} + \gamma_{12} X_{24,t-1} + \delta_3 X_{15,t-1} - \tau_1 X_{16,t-1} - \mu_4 X_{16,t-1}$
$X_{17,t} = X_{17,t-1} + \Theta_1 X_{9,t-1} - \gamma_5 X_{17,t-1} - \Psi_1 X_{17,t-1} - \mu_9 X_{17,t-1}$
$X_{18,t} = X_{18,t-1} + \Theta_2 X_{10,t-1} - \gamma_6 X_{18,t-1} - \Psi_2 X_{18,t-1} - \mu_{10} X_{18,t-1}$
$X_{19,t} = X_{19,t-1} + \Theta_3 X_{11,t-1} - \gamma_7 X_{19,t-1} - \Psi_3 X_{19,t-1} - \mu_{11} X_{19,t-1}$
$X_{20,t} = X_{20,t-1} + \Theta_4 X_{12,t-1} + \tau_1 X_{16,t-1} - \gamma_8 X_{20,t-1} - \Psi_4 X_{20,t-1} - \mu_{12} X_{20,t-1}$
$X_{21,t} = X_{21,t-1} + \Psi_1 X_{17,t-1} - \gamma_9 X_{21,t-1} - \mu_{13} X_{21,t-1}$
$X_{22,t} = X_{22,t-1} + \Psi_2 X_{18,t-1} - \gamma_{10} X_{22,t-1} - \mu_{14} X_{22,t-1}$
$X_{23,t} = X_{23,t-1} + \Psi_3 X_{19,t-1} - \gamma_{11} X_{23,t-1} - \mu_{15} X_{23,t-1}$
$X_{24,t} = X_{24,t-1} + \Psi_4 X_{20,t-1} - \gamma_{12} X_{24,t-1} - \mu_{16} X_{24,t-1}$

$$\begin{aligned} D_t \quad = D_{t-1} \quad & + \mu_1(X_{1,t-1} + X_{5,t-1} + X_{13,t-1}) + \mu_2(X_{2,t-1} + X_{6,t-1} + X_{14,t-1}) + \mu_3(X_{3,t-1} + X_{7,t-1} + X_{15,t-1}) \\ & + \mu_4(X_{4,t-1} + X_{8,t-1} + X_{16,t-1}) + \mu_5 X_{9,t-1} + \mu_6 X_{10,t-1} + \mu_7 X_{11,t-1} + \mu_8 X_{12,t-1} + \mu_9 X_{17,t-1} \\ & + \mu_{10} X_{18,t-1} + \mu_{11} X_{19,t-1} + \mu_{12} X_{20,t-1} + \mu_{13} X_{21,t-1} + \mu_{14} X_{22,t-1} + \mu_{15} X_{23,t-1} + \mu_{16} X_{24,t-1} \end{aligned}$$

Table S1. Model compartments: a legend for the system of equations

Compartment	Description		
S	Susceptible	At risk of acquiring HIV	
D	Dead	Death (absorbing compartment)	
X_1	Infected	Undiagnosed	CD4>500
X_2			CD4 >350 – 500
X_3			CD4 >200 – 350
X_4			CD4 ≤200
X_5		Diagnosed, not in care	CD4>500
X_6			CD4 >350 – 500
X_7			CD4 >200 – 350
X_8			CD4 ≤200
X_9		Linked to care*	CD4>500
X_{10}			CD4 >350 – 500
X_{11}			CD4 >200 – 350
X_{12}			CD4 ≤200
X_{13}		Lost to follow-up†	CD4>500
X_{14}			CD4 >350 – 500
X_{15}			CD4 >200 – 350
X_{16}			CD4 ≤200
X_{17}		On ART, virally suppressed‡	CD4 >500
X_{18}			CD4 >350 – 500
X_{19}			CD4 >200 – 350
X_{20}			CD4 ≤200
X_{21}		On ART, not virally suppressed‡	CD4 >500
X_{22}			CD4 >350 – 500
X_{23}			CD4 >200 – 350
X_{24}			CD4 ≤200

* Defined as HIV care engagement after HIV diagnosis. † Defined as not continuously engaged in HIV care for at least 12 months (pre-ART) or 6 months (on ART). ‡CD4 strata is assumed to be the same as the CD4 at ART initiation.

Table S2. CA-IeDEA HIV policy model parameters

Subscript	Description
r	Sub-population*
j	Partners' sub-population
i	Compartment
t	Time period
**Parameters (force of infection)**	
β_r	Probability of HIV transmission per unprotected sex contact when not suppressed for sub-population *r*
η_r	Average number of sexual acts per month for sub-population *r*
c_r	Percentage of individuals consistently using a condom for sub-population r
ε	Reduction in probability of HIV transmission when using a condom
π	Reduction in probability of HIV transmission when on ART and virally suppressed
$p_{v,\mathrm{j,t}}$	Probability of individuals living with HIV who are on ART and viral suppressed at time period t for sub-population j
$p_{\mathrm{n,j,t}}$	Probability of individuals living with HIV who are not viral suppressed at time period t for sub-population j
N_r	Number of individuals for sub-population *r*
P_{rt}	HIV prevalence at time period t for sub-population *r*
ω_r	Multiplier applied for condom use for sub-population *r*
κ	Multiplier applied for viral suppressed individuals
**Parameters (transition probabilities)†**	
λ_{tr}	Force of infection in sub-population r at time t
α_{it}	Probability of HIV diagnosis for compartment i at time t
δ_i	Probability of HIV disease progression for compartment i
μ_i	Probability of death for compartment i
σ_i	Probability of linkage to care for compartment i
γ_i	Probability of lost to follow-up for compartment i
θ_i	Probability of on ART and virally suppressed for compartment i
τ_i	Probability of return to ART and virally suppressed for compartment i
Ψ_i	Probability of failure to maintain viral suppression for compartment i
ξ	Multiplier applied to adjust probabilities of diagnosis and linkage for population with CD4<200
**Compartments**	
S	Number of susceptible individuals at time t
X_i	Number of infected individuals in compartment i at time t
D	Number of individuals who died at time t

Abbreviations: DHS = Demographic Health Survey; IeDEA=International epidemiology Databases to Evaluate AIDS;
* Sub-populations are defined by age, sex, urbanicity and HIV acquisition risk. † Parameters are for the implemented system of difference equations.

2 Force of infection and mixing

The force of infection (λ_{tr}) of the susceptible population in sub-population r is defined as the probability of the susceptible population acquiring HIV at time t [1]. **Equation 1** presents the force of infection of sub-population r at time period t. Parameters used in the force of infection are defined in Table S2.

$$\Lambda_{r,t} = 1 - \prod_j (1 - \frac{N_j}{\sum_j N_j} \mathrm{P}_{j,t-1} \left(\left((1-\pi) p_{v,j,t-1} + p_{n,j,t-1} \right) \beta \right)^{\eta_r (1 - c_r \varepsilon)}) \quad (1)$$

In the equation, $\lambda_{r,t}$ denotes the probability of HIV acquisition for sub-population r in month t. Π_j is the product of the estimated probabilities of not acquiring HIV when sub-population r partnering with sub-population j. N_j denotes the population size of sub-population j. $\mathrm{P}_{j,t-1}$ denotes HIV prevalence in sub-population j in month *t-1*. c_r represents the proportion of consistent condom use in sub-population r. ε is the effectiveness of consistent condom use. π is the reduction of HIV transmission when virally suppressed. $p_{v,j,t-1}$ and $p_{n,j,t-1}$ denote the proportion of individuals living with HIV that are virally suppressed and not virally suppressed in month *t-1*, respectively. β represents the probability of HIV transmission per unprotected sex act with individuals not virally suppressed. η_r is the monthly number of the sex acts for sub-population *r*.

The pattern for sub-population *r* partnering with sub-population *j* is described by mixing (**Figure S1)**. We assumed that MSM will have sexual intercourse with MSM only, which is consistent with other mathematical models. We assumed no migration between urban and rural areas due to low mobility across regions, except for high-risk women. Finally, we assumed random mixing (i.e., equal chance of partnering regardless of characteristics).

Figure S1. Sub-populations and mixing patterns in the CA-IeDEA model
Legend: Arrows indicate mixing across sub-populations. The probabilities of mixing differ depending on the direction of the arrows. Sub-populations are numbered from 1 to 35 so that they can be identified in model implementation.

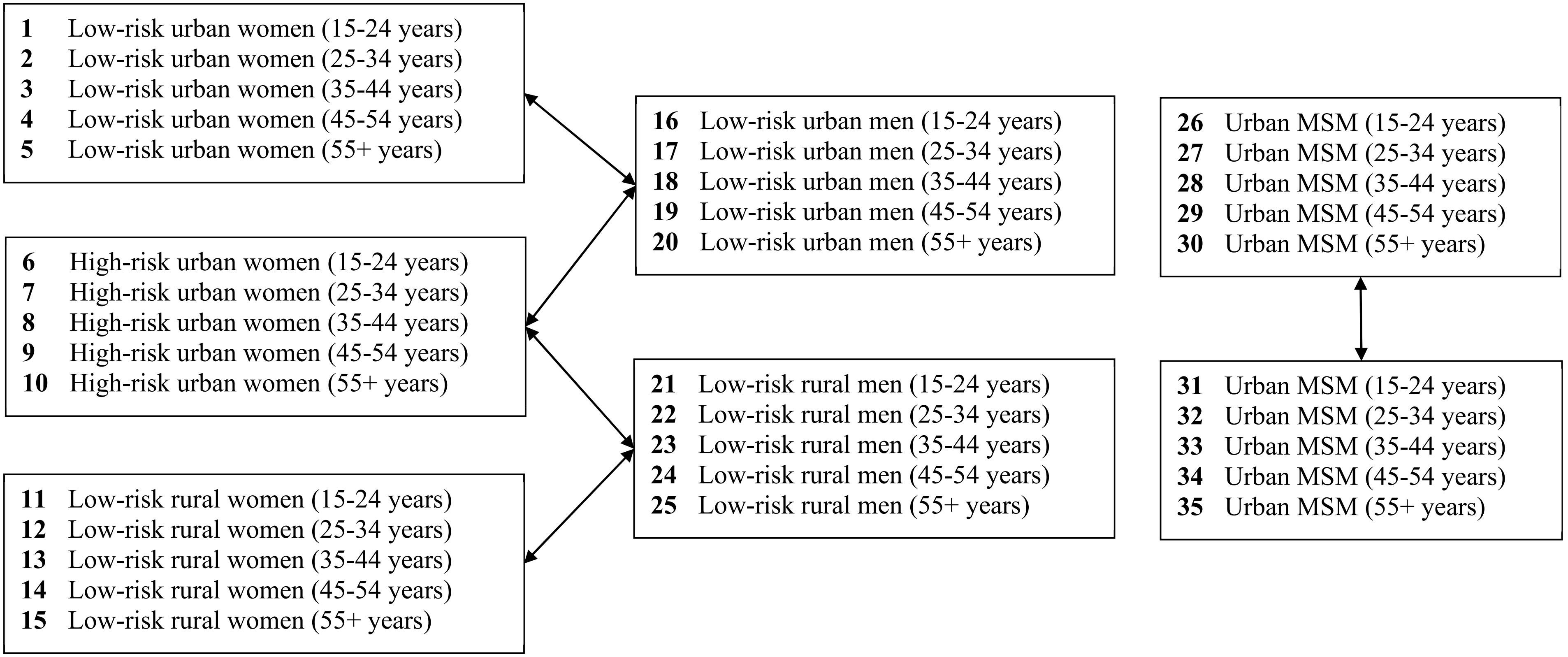

3 Parameterizing the force of infection equation

Probability of HIV transmission per sex act

The probability of acquiring HIV in the force of infection equation is defined as the probability of HIV transmission per sex act in the absence of condom use and partnered with non-virally suppressed partners. A systematic review of probability of HIV transmission per sex act is used (**Table S3)** [2, 3]. We assume that the per-act probability of HIV transmission is the same irrespective of age and urbanicity since a study suggests that the probability of HIV transmission does not statistically differ by the susceptible population's age groups [4].

Condom effectiveness

The effectiveness of condom is defined as the reduction of the probability of HIV transmission per sex act when using condoms consistently. We use a systematic review for condom effectiveness assuming that only male condom will be used [5]. The effectiveness of condom use is applied on a per-act basis (Table S3).

Proportion of consistent condom use

The proportion of consistent condom use is measured as the proportion of individuals in a sub-population that report always using a condom during sexual contacts. We used Demographic Health Surveys (DHS, 2005, 2010, 2015) and the Behavioral and Biological Surveillance Survey (BBSS, 2010, 2020) in Rwanda to estimate the proportion of consistent condom use by sub-populations (**Table S4**) [6-10]. We assume that the proportion of consistent condom use does not differ for population aged 25 years and above since the estimates do not differ significantly. We also assume that the proportion of consistent condom use for high-risk population does not differ by age group or calendar year.

Effectiveness of viral suppression

The effectiveness of viral suppression is defined as the reduction in the probability of HIV transmission per sex act when an individual living with HIV is virally suppressed but not using condoms consistently. Viral suppression status is defined as having HIV RNA viral load < 1000 copies/mL. We estimate the effectiveness of viral suppression in reducing HIV transmission based on existing studies reporting per-act HIV transmission probability by viral suppression status [11] in Equation 2 (see also Table S3):

$$effectiveness = \frac{trans_{all} - trans_{vs}}{trans_{all}} \qquad (2)$$

where $trans_{all}$ denotes per-act transmission probability without ART and $trans_{vs}$ denotes per-act transmission probability when virally suppressed.

Number of sex acts

The number of sex acts is the number of vaginal sex in the past 30 days for both men and women regardless of their partner's HIV status. We use the median number of vaginal sex acts for female sex workers and women who receive voluntary HIV counseling and testing from existing literature in Kigali, Rwanda (Table S3) [12]. Due to lack of data, we assume that the number of sex acts for low-risk women and low-risk men are the same as women who receive voluntary HIV counseling and testing and that the number of sex acts does not differ by age and

urbanicity. Anal sex is not captured given only a small proportion of FSWs engage in anal sex [12]. We utilized the median number of sex partners of MSM to estimate number of sex acts per month [13], assuming stable numbers of sex partners over time and that the number of sex acts is once per partner per month.

Table S3. Probabilities and multipliers for the force of infection, by sub-population and direction

Probabilities	Probabilities (95% CI)	Data source
HIV transmission per sex act		
High-risk female, Male-to-female*	0.0005 (0.0002 – 0.0013)[a]	Boily (2009) [2]
Low-risk female, Male-to-female	0.0030 (0.0014 – 0.0063)	
Male, Female-to-male	0.0087 (0.0028 – 0.0270)	
Male, Male-to-male	0.0082 (0.0024 – 0. 0276)	Vittinghoff (1999) [3]
Effectiveness of condom	80% (34.5% – 94.2%)	Weller (2002) [5]
Proportion of condom use	See Table S4	DHS (2005, 2010, 2015) [6-8], BBSS (2010, 2020) [9, 10]
Effectiveness of viral suppression	96.6% (92.8%, 99.4%)	Supervie (2014) [11]
Number of sex acts		
Low-risk male and female	1 (0–8)	Braunstein (2011) [12]
High-risk female	40 (20, 64)	Braunstein (2011) [12]
MSM	4 (1, 40)	Binagwaho (2010) [13]

Note: 95% CIs are extracted from the literature.
Abbreviations: DHS=Demographic Health Survey; BBSS=Behavioral and Biological Surveillance Survey.
* The probability of client-to-female-sex workers transmission is low possibly due to imprecise data or low prevalence of other sexually transmitted diseases among female sex workers in the country studied.

Table S4. Proportion of consistent condom use, by sub-population

Sub-group	Age group (Years)	Proportion of consistent condom use	Reference
Low-risk urban women	15–24	0.134 (0.091– 0.194)	DHS (Rwanda, 2005) [6]
	25+*	0.043 (0.030 – 0.063)	
	15–24	0.165 (0.123 – 0.218)	DHS (Rwanda, 2010) [7]
	25+*	0.066 (0.050 – 0.085)	
	15–24	0.266 (0.221 – 0.316)	DHS (Rwanda, 2015) [8]
	25+*	0.090 (0.075 – 0.108)	
Low-risk rural women	15–24	0.017 (0.010 – 0.027)	DHS (Rwanda, 2005) [6]
	25+*	0.008 (0.006 – 0.012)	
	15–24	0.066 (0.053 – 0.082)	DHS (Rwanda, 2010) [7]
	25+*	0.032 (0.028 – 0.038)	
	15–24	0.098 (0.082 – 0.117)	DHS (Rwanda, 2015) [8]
	25+*	0.039 (0.034 – 0.045)	
Urban men	15–24	0.408 (0.290 – 0.536)	DHS (Rwanda, 2005) [6]
	25+*	0.120 (0.090 – 0.159)	
	15–24	0.512 (0.407 – 0.617)	DHS (Rwanda, 2010) [7]
	25+*	0.121 (0.096 – 0.153)	
	15–24	0.622 (0.527 – 0.708)	DHS (Rwanda, 2015) [8]
	25+*	0.133 (0.109 – 0.161)	
Rural men	15–24	0.089 (0.060 – 0.131)	DHS (Rwanda, 2005) [6]
	25+*	0.014 (0.009 – 0.020)	
	15–24	0.210 (0.173 – 0.253)	DHS (Rwanda, 2010) [7]
	25+*	0.040 (0.034 – 0.049)	
	15–24	0.276 (0.230 – 0.325)	DHS (Rwanda, 2015) [8]
	25+*	0.050 (0.042 – 0.058)	
High-risk urban women	15–24†	0.351 (0.298 – 0.404)	BBSS 2010 [9]
	25+*†	0.276 (0.235 – 0.317)	
MSM	15–24†	0.404 (0.343 – 0.465)	BBSS 2020 [10]
	25+*†	0.404 (0.343 – 0.465)	

* We assume the proportion of consistent condom use is the same for individuals aged 25+ years given existing data.

† Due to lack of data, we assume that the confidence interval is a relative ∓15% of the baseline value.

4 Model initialization

To instantiate the model, we use data from multiple sources to estimate the population size for each sub-population across compartments.

The size of sub-populations, by HIV status (Table S5)

We estimate the size of each sub-population using data from the World Bank (low-risk populations) and the 2012 Sex Worker Size Estimation (high-risk urban women) [14, 15], and literature [16]. For low-risk populations, the World Bank reports the population data by age group and sex together with the percent of population living in urban and rural areas. We assume similar age and sex distributions in urban and rural areas to estimate the size of the low-risk sub-populations. For the high-risk populations, BBSS (2010) and the literature report the age-distribution of female sex workers and MSM [17]. While the age-group stratification differs between the BBSS (2010) and our model, we assume uniform distributions within each age-group to derive our own estimates.

We further stratify each sub-population by HIV status using HIV prevalence derived from DHS (2005) (low-risk population) and BBSS (2010, 2020) (high risk population) [6, 9, 10]. We estimate the size of each sub-population living with HIV by multiplying the size of each sub-population and corresponding HIV prevalence. We estimate the size of each susceptible population by subtracting the people with HIV from the size of total population in each sub-population.

Table S5. Initial population size, by sub-population and HIV status

Sub-population	Total population (2003), n	HIV prevalence,* %	People with HIV, n	Susceptible population, n
Low-risk urban women (15–24)	174,420	4.04	7,047	167,373
Low-risk urban women (25–34)	96,551	9.43	9,105	87,446
Low-risk urban women (35–44)	69,640	19.13	13,322	56,318
Low-risk urban women (45–54)	45,588	7.57	3,451	42,137
Low-risk urban women (55–64)†	25,313	7.57	1,916	23,397
Low-risk rural women (15–24)	857,407	0.99	8,488	848,919
Low-risk rural women (25–34)	474,621	3.49	16,564	458,057
Low-risk rural women (35–44)	342,332	4.46	15,268	327,064
Low-risk rural women (45–54)	224,101	3.58	8,023	216,078
Low-risk rural women (55–64)†	124,433	3.58	4,455	119,978
Urban men (15–24)	163,133	1.1	1,794	161,339
Urban men (25–34)	89,208	7.33	6,539	82,669
Urban men (35–44)	72,332	12.35	8,933	63,399
Urban men (45–54)	41,887	7.98	3,343	38,544
Urban men (55–64)	19,325	0	0	19,325
Rural men (15–24)	801,925	0.27	2,165	799,759
Rural men (25–34)	438,524	1.62	7,104	431,420
Rural men (35–44)	355,569	3.61	12,836	342,733
Rural men (45–54)	205,905	2.79	5,745	200,160
Rural men (55–64)	94,995	0	0	94,995
High-risk women (15–24) ‡	5,648	39.5	2,231	3,417
High-risk women (25–34) ‡	4,543	53	2,408	2,135
High-risk women (35–44) ‡	1,498	62	929	569
High-risk women (45–54) ‡	295	63	186	109
High-risk women (55–64) †‡	295	63	186	109
MSM (15–24)	8,996	7	630	8,366
MSM (25–34)	7,548	7	528	7,020
MSM (35–44)	525	7	37	488
MSM (45–54)	525	7	37	488
MSM (45–54)	525	7	37	488

* HIV prevalence data are based on the DHS 2005 data for low-risk population and BSS 2010 for high-risk women.

† Given limited data, we assume that women aged 55+ years have the same HIV prevalence as women aged 45–54 years.

‡ We assume the total population of female sex worker is 12,278 based on the 2012 Sex Worker Size Estimation Survey. The distribution of FSWs by age is estimated based on 2010 BSS data. Given 2010 BSS data has different age groups over 30, we assume a uniform distribution within age groups.

)

The size of sub-populations living with HIV across compartments

The distribution of people with HIV in each sub-population across compartments are estimated first along the HIV care continuum and by CD4 stratum. We use DHS data [6], literature [12], and simple descriptive statistics from IeDEA to estimate the proportion of population along the HIV care continuum and by CD4 strata (**Table S6**). Since we only include individuals enrolled in IeDEA after the starting year, we assume no loss to follow-up at model initiation. Due to the small sample size and limited information in the literature, we assume that the initial distribution of sub-populations across infectious compartments does not differ by sub-populations.

Table S6. Parameters to initialize the model

**Parameters**	**Baseline values**	**Data source**
Proportion diagnosed among all people with HIV	44.3% (30.6%, 50.0%)	DHS (Rwanda, 2005) [6]
Proportion linked among people diagnosed with HIV	85%	Braunstein (2011) [12]
Proportion on ART and virally suppressed among people linked to care	48.6%	CA-IeDEA (Rwanda, 2004) [18]
Proportion viral failure among people on ART	25%	Assumed
**CD4 distribution, undiagnosed, diagnosed, LTFU**		
CD4>500	34.0%	Nsanzimana (2015) [19]
CD4 351 – 500	25.3%	
CD4 201 – 350	27.2%	
CD4<200	13.5%	
**CD4 distribution, linked**		
CD4>500	39.1%	CA-IeDEA (Rwanda, 2004) [18]
CD4 351 – 500	18.0%	
CD4 201 – 350	21.1%	
CD4<200	21.8%	
**CD4 distribution, on ART and suppressed**		
CD4>500	0	CA-IeDEA (Rwanda, 2004) [18]
CD4 351 – 500	4.8%	
CD4 201 – 350	23.8%	
CD4<200	71.4%	
**CD4 distribution before linkage**		
CD4>500	0	CA-IeDEA (Rwanda, 2004) [18]
CD4 351 – 500	0	
CD4 251 – 350	0	
CD4<200	100%	

Abbreviations: DHS=Demographic Health Survey; CA-IeDEA= Central Africa International epidemiology Databases to Evaluate AIDS

Susceptible population growth rate

We apply a monthly growth rate for each sub-population. Data come from World Bank annual population estimates and projections, 2004–2045 [15]. We estimate the average monthly population growth rate overtime regardless of HIV status as a proxy for the growth rate of susceptible population (**Table S7**). We make several assumptions in estimating the population growth for both low-risk and high-risk sub-populations. The population growth rate in high-risk female is the same as low-risk female due to lack of data. We assume constant population growth rate overtime. Based on the data, we assume that the growth rate for the susceptible population aged 25–34, 35–44, and 45–54 years with similar sex, risk status, and residency does not vary since the growth rates for susceptible population within this age group are not statistically different. Due to data limitation, we assume the population growth rate for MSM is the same as for low-risk men.

Table S7. Average monthly growth rate for sub-populations*

**Sub-population**	**Monthly growth rate**	**95% Confidence Interval**
Low-risk urban women (15–24)	0.20%	(0.17%, 0.23%)
Low-risk urban women (25–54) †	0.33%	(0.32%, 0.34%)
Low-risk urban women (55–64)	0.45%	(0.40%, 0.50%)
Low-risk rural women (15–24)	0.08%	(0.05%, 0.11%)
Low-risk rural women (25–54) †	0.21%	(0.19%, 0.24%)
Low-risk rural women (55–64)	0.33%	(0.29%, 0.37%)
Urban men (15–24)	0.21%	(0.18%, 0.24%)
Urban men (25-54) †	0.33%	(0.31%, 0.34%)
Urban men (55–64)	0.48%	(0.42%, 0.53%)
Rural men (15–24)	0.09%	(0.06%, 0.12%)
Rural men (25–54) †	0.21%	(0.19%, 0.24%)
Rural men (55–64)	0.36%	(0.30%, 0.41%)
High-risk urban women (15–24) ‡	0.20%	(0.17%, 0.23%)
High-risk urban women (25–54) †‡	0.33%	(0.32%, 0.34%)
High-risk urban women (55–64) ‡	0.45%	(0.40%, 0.50%)

* The monthly growth rate is estimated based on the World Bank population estimates and projection [15]. The monthly population growth rate is an average of the monthly growth rate between 2003 and 2045.

† We adopt the same population growth rate for individuals aged 25–34, 35–44, and 45–54 years because previous analysis showed that the growth rate within the three age groups did not differ significantly.

‡ We assume that the population growth rate in high-risk urban women is the same as low-risk urban women.

5 Transition probabilities

5.1 HIV diagnosis

We use the proportion of population receiving an HIV test within the past 12 months as the probability of HIV diagnosis, which is consistent with other modelling studies [20]. We use Demographic and Health Surveys (DHS) for Rwanda in 2005, 2010 and 2015, a national household survey, to estimate the probability of HIV diagnosis for low-risk women and men in both urban and rural areas [6-8]. In the DHS, respondents are asked if they have previously received an HIV test and if so, the timing of the tests. They are also asked whether they have received the result of their last HIV test. A random sub-sample of these respondents receive an HIV test, regardless of prior test history. The BBSS (2010), a survey of female sex workers in Rwanda, is used to estimate the probability of HIV diagnosis among high-risk women in urban areas [9]. In the BBSS (2010), respondents are asked if they have received an HIV test within the past 12 months and if so, has the result been received. We used the literature to identify the proportion of HIV diagnosis within the past 12 months for MSM [21].

We have made several assumptions to estimate the probability of HIV diagnosis:

- The probability of HIV diagnosis among low-risk populations does not differ by urbanicity, given no statistically significant difference in our estimates for urban versus rural populations (2010 and 2015 only).
- The probabilities of HIV diagnosis for men in age groups 15–24 years and 25–34 years do not differ, and the probabilities of HIV diagnosis for men and women in age groups 35–44, 45-44, and 55+ do not differ. The assumption is made given small sub-population sample sizes and similar estimates across select sub-populations.
- The probability of HIV diagnosis vary for individuals with CD4≤200, given the evidence suggesting those with CD4 count ≤200 are 1.56 times (95% CI 1.11, 2.20) more likely to be linked compared to those with CD4>200 [22]. The evidence is applicable to HIV diagnosis since it is comparable with the estimates used in other modelling studies [23].
- For low-risk sub-populations, respondents who receive their last HIV test result more than 12 months prior to the DHS interview date know their HIV status.
- For high-risk sub-populations, the probability of HIV diagnosis is the same regardless of HIV status, since high-risk sub-populations are targeted by HIV testing promotion policies and campaigns [24].
- For high-risk sub-populations, the probability of HIV diagnosis does not vary based on age and urbanicity, given similar estimates in the sample by age and region [9].

Table S8. Monthly probability of HIV diagnosis, by sub-population and CD4 stratum

Sub-population	Years of data applied	Age group (Years)	Monthly probabilities (95% CI)		Reference
			CD4 >200*	CD4 ≤200	
Low-risk women	2004 – 2005	15–24	0.016 (0.007 – 0.037)	0.025 (0.010 – 0.057)	DHS (Rwanda, 2005) [6]
		25–34	0.027 (0.016 – 0.043)	0.041 (0.025 – 0.067)	
		35–44	0.027 (0.018 – 0.042)	0.043 (0.027 – 0.065)	
		45–54	0.027 (0.018 – 0.042)	0.043 (0.027 – 0.065)	
		55+	0.027 (0.018 – 0.042)	0.043 (0.027 – 0.065)	
	2006 – 2010	15–24	0.145 (0.058 – 0.246)	0.226 (0.091 – 0.383)	DHS (Rwanda, 2010) [7]
		25–34	0.146 (0.076 – 0.225)	0.228 (0.118 – 0.351)	
		35–44	0.111 (0.069 – 0.160)	0.173 (0.108 – 0.250)	
		45–54	0.111 (0.069 – 0.160)	0.173 (0.108 – 0.250)	
		55+	0.111 (0.069 – 0.160)	0.173 (0.108 – 0.250)	
	2011 – End of projection period	15–24	0.126 (0.065 – 0.197)	0.196 (0.101 – 0.307)	DHS (Rwanda, 2015) [8]
		25–34	0.133 (0.081 – 0.191)	0.207 (0.127 – 0.297)	
		35–44	0.115 (0.066 – 0.173)	0.179 (0.103 – 0.269)	
		45–54	0.115 (0.066 – 0.173)	0.179 (0.103 – 0.269)	
		55+	0.115 (0.066 – 0.173)	0.179 (0.103 – 0.269)	
Low-risk men	2004 – 2005	15–24	0.018 (0.008 – 0.038)	0.028 (0.012 – 0.059)	DHS (Rwanda, 2005) [6]
		25–34	0.018 (0.008 – 0.038)	0.028 (0.012 – 0.059)	
		35–44	0.021 (0.012 – 0.037)	0.033 (0.018 – 0.058)	
		45–54	0.021 (0.012 – 0.037)	0.033 (0.018 – 0.058)	
		55+	0.021 (0.012 – 0.037)	0.033 (0.018 – 0.058)	
	2006 – 2010	15–24	0.071 (0.024 – 0.226)	0.111 (0.038 – 0.353)	DHS (Rwanda, 2010) [7]
		25–34	0.071 (0.024 – 0.226)	0.111 (0.038 – 0.353)	
		35–44	0.139 (0.076 – 0.210)	0.217 (0.119 – 0.328)	
		45–54	0.139 (0.076 – 0.210)	0.217 (0.119 – 0.328)	
		55+	0.139 (0.076 – 0.210)	0.217 (0.119 – 0.328)	
	2011 – End of projection period	15–24	0.045 (0.037 – 0.111)	0.071 (0.058 – 0.173)	DHS (Rwanda, 2015) [8]
		25–34	0.045 (0.037 – 0.111)	0.071 (0.058 – 0.173)	
		35–44	0.146 (0.098 – 0.198)	0.228 (0.153 – 0.309)	
		45–54	0.146 (0.098 – 0.198)	0.228 (0.153 – 0.309)	
		55+	0.146 (0.098 – 0.198)	0.228 (0.153 – 0.309)	
High-risk urban women	2004 – End of projection period	15–24	0.168 (0.088 – 0.241)	0.241 (0.129 – 0.339)	BBSS 2010 [9]
		25–34	0.168 (0.088 – 0.241)	0.241 (0.12 – -0.339)	
		35–44	0.168 (0.088 – 0.241)	0.241 (0.129 – 0.339)	
		45–54	0.168 (0.088 – 0.241)	0.241 (0.129 – 0.339)	
		55+	0.168 (0.088 – 0.241)	0.241 (0.129 – 0.339)	
MSM	2004 – End of projection period	15-55+	0.113 (0.096 – 0.130)	0.176 (0.149 – 0.203)	Ntale (2019) [21]

* CD4 >200 represents the following CD4 strata: >500, >350–500, and >200–350.

5.2 Natural history disease progression

The natural history of HIV is defined as advancing through CD4 count strata (>500, >350–500, >200–350, ≤200). To estimate the natural history of HIV, we use the Rwanda cohort of the Central Africa International epidemiology databases to evaluate AIDS (CA-IeDEA) consortium from 2004 to 2020. We use ART-naïve individuals with at least 2 pre-ART CD4 tests residing in districts with non-missing death information as our sample. Natural history disease progression is estimated using incidence density analysis. The annual rate for natural history disease progression is calculated as the number of events (i.e., reaching a given CD4 threshold) divided by the total time at risk within a given CD4 stratum. The total time at risk is defined as the total residence time within a CD4 stratum before the event occurs assuming a linear trend of changes in CD4 values. The incidence rate is converted to a monthly probability so that it aligns with our model cycle.

Table S9. Monthly probabilities of natural history disease progression

	CD4 >500	CD4 350-500	CD4 200-350
Male	0.026 (0.025, 0.027)	0.015 (0.014, 0.023)	0.057 (0.057, 0.059)
Female	0.008 (0.007, 0.009)	0.019 (0.018, 0.020)	0.047 (0.044, 0.048)

5.3 Linkage to care

To inform linkage to care, we use a cross-sectional study that collects HIV care and treatment information from individuals who participated in a national HIV care program, 2013–2014, in Rwanda [25]. For high-risk urban women, we use data from a cohort study examining the rate of linkage and treatment since diagnosis in 2007–2008 [12]. Due to lack of data, we assume that the probabilities of linkage to care are the same for individuals in CD4 count strata >200–350, >350–500, and >500. Linkage to care for those with CD4≤200 is 1.56 times more likely to occur compared to those with CD4>200. This assumption is based on a randomized controlled trial in Kenya that examined testing and linkage to care based on CD4, with 1.56 (95% CI 1.11-2.20) times vs those with CD4>200 [22]. We also assume that linkage to care is the same irrespective of sex, age and urbanicity due to limited data. The annual probability of linkage to care is converted to a monthly probability. Since the original data does not provide the confidence interval, we use 0.5 and 1.5 times the baseline value as the lower and upper bound, respectively, of the confidence interval (**Table S10**). Due to lack of data for MSM, we assumed that the probability of linkage is similar to baseline as for high-risk women but allow for wider variation using the upper bound and lower bound from both the high- and low-risk populations.

Table S10. Monthly probability of sub-population linkage to HIV care, by CD4 stratum

Sub-population	Monthly probability (95% CI)		Reference
	CD4>500, CD4>350–500, CD4>200–350	CD4≤200	
High-risk women	0.115 (0.059 – 0.168)	0.174 (0.091 – 0.249)	Nsanzimana (2015) [25]
Low-risk women and men	0.175 (0.092 – 0.250)	0.259 (0.139 – 0.362)	Braunstein (2011) [12]
MSM	0.115 (0.059 – 0.250)	0.174 (0.091 – 0.362)	Assumption

5.4 Lost to follow-up

LTFU is defined as at least 365 days between the last clinical visit and the censor date before ART initiation or as at least 6 months between last clinical visit and the censor date when on ART (**Table S11**). The definition is based on Rwanda national HIV treatment guidelines and is consistent with other studies examining LTFU in sub-Saharan Africa [26]. We do not stratify the analysis (see below) by sub-population due to the small sample size. Similar to natural history disease progression, we use CA-IeDEA data and apply incidence density analysis to estimate the probability of LTFU. Specifically, we adjust for misclassification of LTFU in the data. Evidence from sub-Saharan Africa suggests that some patients recorded as LTFU have died or transferred to another clinic [27-31]. After estimating the probability of LTFU, we adjust for mortality and self-transfer among patients misclassified as LTFU using a multiplier [29].

On ART, Suppressed

Patients who are on ART and virally suppressed are assumed to be those on ART for at least 6 months and without evidence of virological failure. Virological failure is defined as HIV RNA >1000 copies/ml based on Rwanda HIV guidelines [32]. We use CA-IeDEA data and incidence density analysis to estimate the probability on ART and virally suppressed. Parameter values for the probability of on ART and virally suppressed vary based on disease progression and year **(Table S11**). Model compartments that apply to probability of on ART and virally suppressed include only linkage to care.

On ART, Not Suppressed

We define transition from on ART and suppressed to on ART and not suppressed as virologic failure. Virologic failure is defined as virally suppressed patients with at least 1 viral load test of >1000 HIV RNA copies/ml in the follow-up tests based on Rwanda HIV guidelines to account for differences in the probability of HIV transmission [32]. We use CA-IeDEA data and incidence density analysis to estimate the probability on ART and not virally suppressed. Parameter values of probabilities of on ART and not virally suppressed (or virological failure) vary based on disease progression (Table S11). Due to limited viral load test data, we assume that a single HIV RNA test result of >1000 copies/ml indicates virologic failure.

Table S11. Monthly probabilities of LTFU, on ART and suppressed, and viral failure

	**CD4 >500**	**CD4 350–500**	**CD4 200–350**	**CD4 <200**
LTFU				
Pre-ART	0.012 (0.011, 0.013)	0.008 (0.007, 0.009)	0.012 (0.010, 0.014)	0.018 (0.014, 0.022)
On-ART	0.012 (0.011, 0.013)	0.010 (0.009, 0.011)	0.013 (0.012, 0.014)	0.017 (0.016, 0.018)
On ART and suppressed				
2006 and before	0.002 (0.001, 0.002)	0.003 (0.001, 0.004)	0.018 (0.014, 0.022)	0.096 (0.088, 0.104)
2007-2011	0.005 (0.004, 0.005)	0.010 (0.009, 0.012)	0.061 (0.057, 0.066)	0.125 (0.117, 0.133)
2012-2015	0.013 (0.012, 0.015)	0.027 (0.024, 0.030)	0.082 (0.074, 0.090)	0.119 (0.108, 0.130)
2016 and after	0.089 (0.082, 0.097)	0.135 (0.120, 0.150)	0.144 (0.128, 0.160)	0.148 (0.131, 0.164)
On ART and not suppressed				
All years	0.002 (0.001, 0.003)	0.001 (0.001, 0.002)	0.001 (0.001, 0.002)	0.002 (0.001, 0.003)

5.5 Death

We define probability of death as all-cause mortality (i.e., HIV-related and non HIV-related death). We use CA-IeDEA data and incidence density analysis to estimate the probability of death. Enrollees are considered having died if they are classified as dead in the CA-IeDEA database. The parameter estimates for probability of death apply to all infectious compartments, including Undiagnosed, Diagnosed, Linkage to care, Loss-to follow up (LTFU), On ART and suppressed, and On ART and not suppressed. We assume that the probability of death for undiagnosed, diagnosed, and LTFU compartments are similar to the probability of death for linked to care but not on ART due to limited data.

Table S12. Annual probability of sub-population death, by CD4 stratum and care engagement*

Sub-population	On ART & suppressed			
	**CD4 >500**	**CD4 350–500**	**CD4 200–350**	**CD4 <200**
Female (15–24)	0.0025<br>(0.0001, 0.0343)	0.0117<br>(0.0024, 0.0343)	0.0148<br>(0.0018, 0.0534)	0.0245<br>(0.0050, 0.0715)
Female (25–34)	0.0062<br>(0.0020, 0.0145)	0.0060<br>(0.0020, 0.0141)	0.0021<br>(0.0001, 0.0534)	0.0139<br>(0.0038, 0.0356)
Female (35–44)	0.0032<br>(0.0001, 0.0180)	0.0115<br>(0.0024, 0.0335)	0.0144<br>(0.0047, 0.0338)	0.0126<br>(0.0034, 0.0320)
Female (45–54)	0.0141<br>(0.0004, 0.0788)	0.0320<br>(0.0087, 0.0819)	0.0099<br>(0.0003, 0.1252)	0.0099<br>(0.0003, 0.1252)
Female (55–64)	0.0050<br>(0.0001, 0.279)	0.0178<br>(0.0037, 0.0521)	0.0225<br>(0.0006, 0.1252)	0.2019<br>(0.0006, 0.7294)
Male (15-19)	0.0082<br>(0.0002, 0.0397)	0.0137<br>(0.0004, 0.0762)	0.0197<br>(0.0005, 0.1100)	0.0593<br>(0.0175, 0.1455)
Male (25–34)	0.0040<br>(0.0005, 0.0143)	0.0056<br>(0.0012, 0.0165)	0.0076<br>(0.0016, 0.0222)	0.0297<br>(0.0136, 0.0563)
Male (35–44)	0.0034<br>(0.0001, 0.0191)	0.0078<br>(0.0021, 0.0201)	0.0095<br>(0.0031, 0.0221)	0.0232<br>(0.0100, 0.0457)
Male (45–54)	0.0159<br>(0.0002, 0.0918)	0.0362<br>(0.0133, 0.0787)	0.0252<br>(0.0092, 0.0548)	0.0095<br>(0.0012, 0.0343)
Male (55–64)	0.1000<br>(0.0001, 0.0191)	0.0255<br>(0.0006, 0.1420)	0.0228<br>(0.0021, 0.1006)	0.0394<br>(0.0010, 0.2193)
Multiplier for pre-ART	7.14<br>(3.33, 14.29)	7.14<br>(3.33, 14.29)	7.14<br>(3.33, 14.29)	7.14<br>(3.33, 14.29)

* The probability of death for individuals undiagnosed, diagnosed, LTFU, and not suppressed will be the same, calculated as on ART mortality times the multiplier.

6 Calibration targets

We identify 47 calibration targets based on Rwanda national surveys and reports (**Table S14** – **Table S16**) [33-36]. The targets include: 1) HIV prevalence; 2) HIV incidence; 3) number of individuals on ART; 4) percentage of individuals diagnosed with HIV on ART; 5) percent virally suppressed; and 6) percent virally suppressed conditional on ART. The calibration targets are presented below.

Table S13. HIV epidemic targets, by sub-population

Target	Age Group	2005	2010	2015	2019
HIV prevalence, % (95% confidence interval)					
Men	15 – 49	2.22 (1.82, 2.70)	2.43 (2.07, 2.86)	2.44 (2.07, 2.87)	1.80 (1.50, 2.10)
	15 – 64	NA	NA	NA	2.20 (1.90, 2.60)
Women	15 – 49	3.56 (3.13, 4.12)	3.71 (3.30, 4.18)	3.62 (3.19, 4.10)	3.30 (2.90, 3.80)
	15 – 64				3.70 (3.30, 4.10)
Overall	15 – 49	2.98 (2.66, 3.33)	3.11 (2.82, 3.42)	3.06 (2.77, 3.38)	2.60 (2.30, 2.90)
	15 – 64	NA	NA	NA	3.00 (2,70, 3.30)
HIV incidence, per 1,000 population (95% confidence interval)					
Men	15 – 49	NA	NA	NA	1.00 (0.00, 2.00)
Women	15 – 49	NA	NA	NA	0.60 (0.00, 1.30)
Overall	15 – 49	NA	NA	NA	0.80 (0.20, 1.40)

Abbreviation: NA=Not available

Table S14. HIV care continuum targets, 15–49 years

Target	Female	Male	Total
Percent known HIV status, % (95% CI)	85.0 (81.2, 88.9)	76.6 (69.4, 83.8)	82.2 (78.3, 86.2)
Percent on ART among diagnosed, % (95% CI)	97.2 (95.5, 98.8)	96.2 (93.2, 99.3)	96.9 (95.3, 97.2)
Percent viral suppressed among ART, % (95% CI)	92.6 (89.9, 95.2)	83.1 (75.9, 90.3)	89.7 (86.7, 92.7)
Percent viral suppressed among overall living with HIV, % (95% CI)	76.5 (72.1, 80.8)	61.3 (52.6, 70.0)	71.5 (66.8, 76.1)
Percent viral suppressed among overall living with HIV, 15-64 years % (95% CI)	77.2 (73.1, 81.4)	66.8 (59.7, 74.0)	73.5 (69.8, 77.8)

Table S15. Number of people on ART, 15–49 years

Year[a]	Number of people with HIV on ART
2004	4,004
2005	12,007
2006	21,506
2007	30,283
2008	39,784
2009	48,337
2010	52,316
2011	60,557
2012	68,001
2013	77,804
2014	82,156
2015	93,530
2016	105,095
2017	110,476
2018	115,282
2019	120,799
2020	124,453

* From 2004 to 2013, the numbers of people with HIV on ART come from TRACnet, a national phone-based and internet-based HIV reporting system in Rwanda [10]. The data are reported in the Rwanda Annual Report for HIV. From 2014 to 2020, the numbers come from the Rwanda annual HIV and hepatitis report. All targets are adjusted to estimate the population aged 15–49 years based on the age-distribution of people on ART reported by Rwanda [10].

Supplemental Results

Additional calibration figures for sub-populations are reported in **Fig S2 2–S3** for HIV epidemic targets and HIV care continuum targets.

Fig S2. Fit of model projections to HIV prevalence, 15–64 years

Abbreviation: PHIA=Population-based Health Impact Assessment

The points and the error bar represent the point estimates and 95% confidence interval of the calibration targets. Calibration targets from targets from PHIA are presented in red. The blue line and light blue shaded region represent the mean and range of the model projections.

Panel A. Total **Panel B. Women** **Panel C. Men**

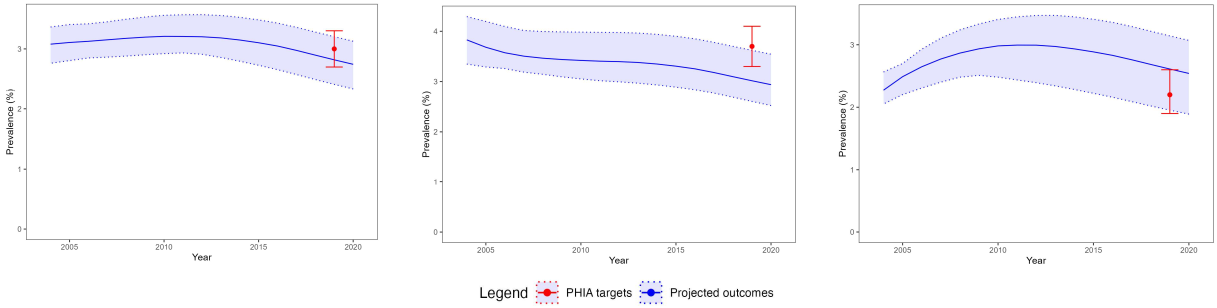

Fig S3. Fit of model projections to HIV care engagement targets, by sex 15–44 years

Abbreviations: DHS=Demographic Health Survey; PHIA=Population-based Health Impact Assessment; ART=Antiretroviral therapy

The red points and the error bar represent the point estimates of the HIV care continuum targets and 95% confidence intervals. The blue line is the mean of the projected HIV care continuum targets among the 50 best-fitting trials; the light blue shaded region is the range of the projected HIV care continuum targets.

Panel A. Women, 15–44 years

Percent diagnosed

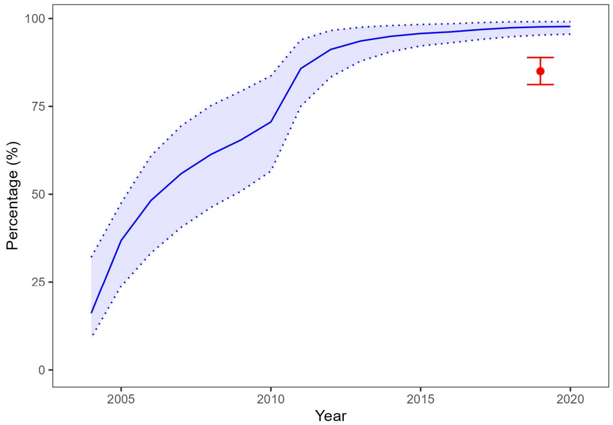

Percent on ART among diagnosed

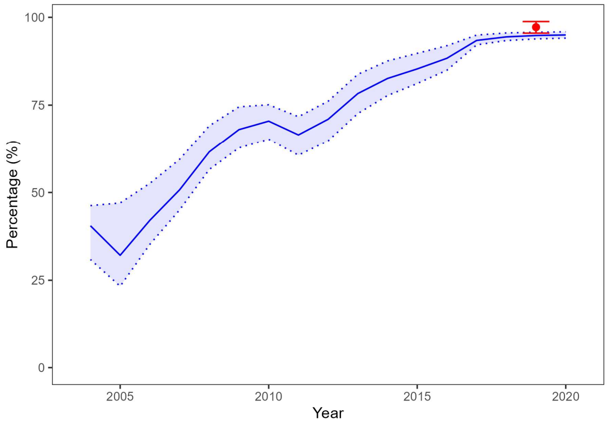

Percent virally suppressed among on ART

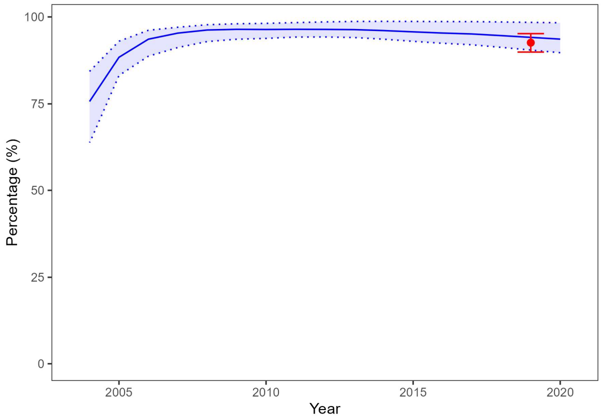

Percent virally suppressed among those with HIV

Panel B. Men, 15–44 years

Percent diagnosed

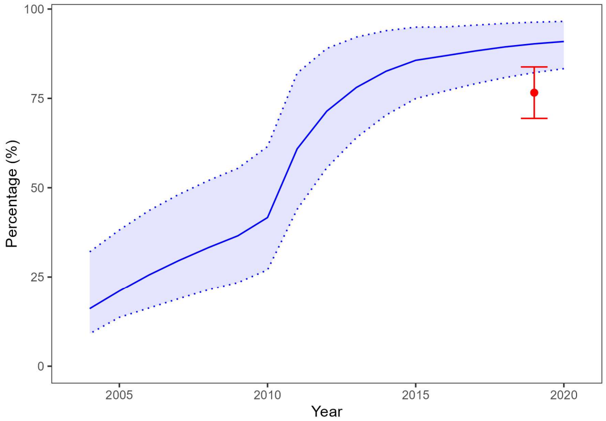

Percent on ART among diagnosed

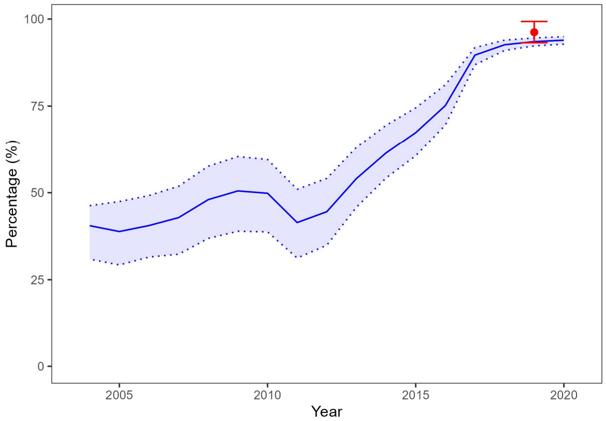

Percent virally suppressed among on ART

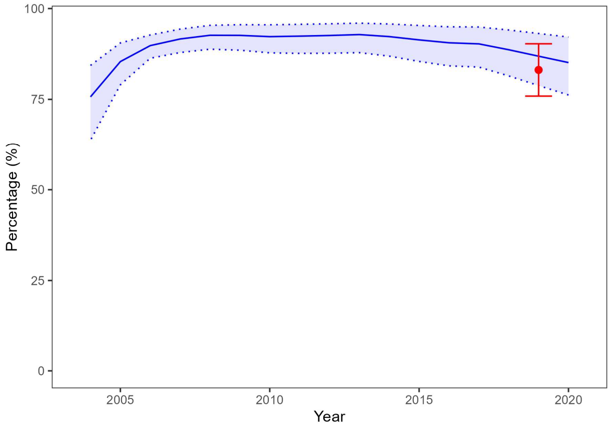

Percent virally suppressed among those with HIV

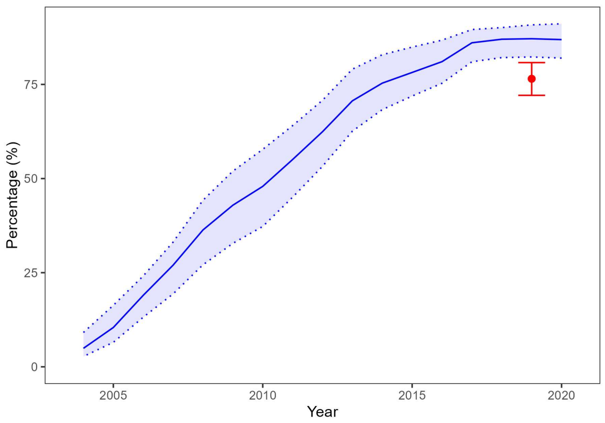

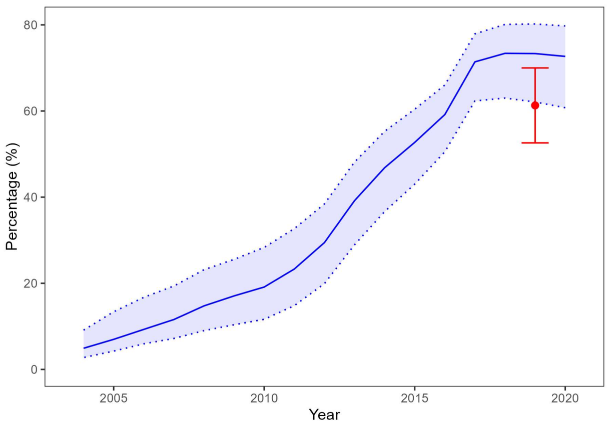

Percent virally suppressed among those with HIV (15–64 years)

Percent virally suppressed among those with HIV (15–64 years)

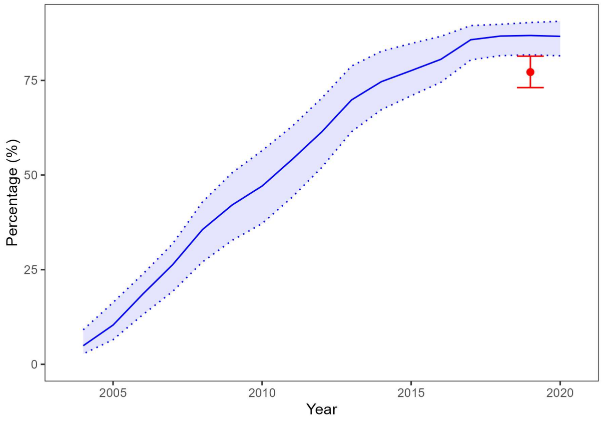

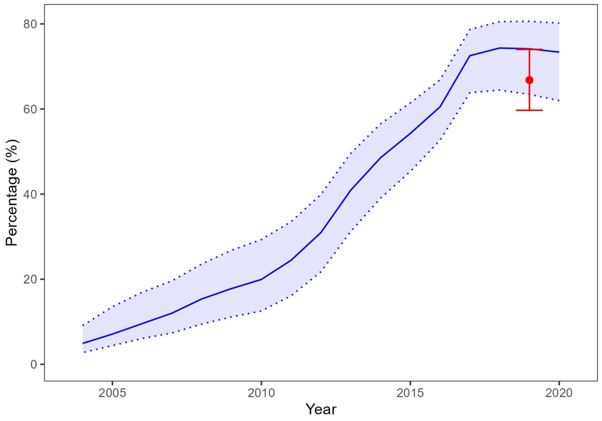

Legend PHIA targets Projected outcomes

References

1. Kerr CC, Stuart RM, Gray RT, Shattock AJ, Fraser-Hurt N, Benedikt C, et al. Optima: a model for HIV epidemic analysis, program prioritization, and resource optimization. J Acquir Immune Defic Syndr. 2015;69(3):365-76.
2. Boily M-C, Baggaley RF, Wang L, Masse B, White RG, Hayes RJ, et al. Heterosexual risk of HIV-1 infection per sexual act: systematic review and meta-analysis of observational studies. Lancet Infect Dis. 2009;9(2):118-29.
3. Vitinghoff E, Douglas J, Judon F, McKiman D, MacQueen K, Buchinder SP. Per-contact risk of human immunodificiency virus tramnsmision between male sexual partners. American journal of epidemiology. 1999;150(3):306-11.
4. Powers KA, Poole C, Pettifor AE, Cohen MS. Rethinking the heterosexual infectivity of HIV-1: a systematic review and meta-analysis. Lancet Infect Dis. 2008;8(9):553-63.
5. Weller SC, Davis-Beaty K. Condom effectiveness in reducing heterosexual HIV transmission. Cochrane Database Syst Rev. 2002;(1):CD003255.
6. Institut National de la Statistique (INSR) [Rwanda], ORC Macro. Rwanda Demographic and Health Survey 2005 [Dataset] Calverton, Maryland, USA: Institut National de la Statistique and ORC Macro [Producers]; ICF [Distributor], 2006 [cited 2020 Febuary 24]. Available from: http://dhsprogram.com/pubs/pdf/FR183/FR183.pdf.
7. National Institute of Statistics (NISR) [Rwanda], Ministry of Health (MOH) [Rwanda], ICF International. Rwanda Demographic and Health Survey 2010 [Dataset] Calverton, Maryland, USA: National Institute of Statistics, Ministry of Health, ICF International [Producers]; ICF [Distributor], 2012 [cited 2020 August 24]. Available from: http://dhsprogram.com/pubs/pdf/FR259/FR259.pdf.
8. National Institute of Statistics [Rwanda], Ministry of Finance and Economic Planning[Rwanda], Ministry of Health[Rwanda], ICF International. Rwanda Demographic and Health Survey 2014-15 [Dataset] Kigali, Rwanda: National Institute of Statistics, Ministry of Finance and Economic Planning, Ministry of Health, and ICF International [Producers]; ICF [Distributor], 2016 [cited 2024 August 24]. Available from: http://dhsprogram.com/pubs/pdf/FR316/FR316.pdf.
9. Rwanda Ministry of Health. Behavioral and biological surveillance survey among female sex workers, Rwanda – 2010: Suevey report 2010 [cited 2024 July 3]. Available from: https://rbc.gov.rw/IMG/pdf/behavioral_and_biological_survey_among_female_sex_workers_rwanda_2010.pdf.
10. Rwanda Ministry of Health. National HIV and viral hepatitis annual report 2020-2021 2021 [cited 2024 July 3]. Available from: https://www.rbc.gov.rw/index.php?id=693.
11. Supervie V, Viard JP, Costagliola D, Breban R. Heterosexual Risk of HIV Transmission per Sexual Act Under Combined Antiretroviral Therapy: Systematic Review and Bayesian Modeling. 2014;59(1):115-22.
12. Braunstein SL, Umulisa MM, Veldhuijzen NJ, Kestelyn E, Ingabire CM, Nyinawabega J, et al. HIV diagnosis, linkage to HIV Care, and HIV risk behaviors among newly diagnosed HIV-positive female sex workers in Kigali, Rwanda. J Acquir Immune Defic Syndr. 2011;57(4):70-6.
13. Binagwaho A, Chapman J, Koleros A, Utazirubanda Y, Pegurri E, Gahire R. Exploring HIV Risk among MSM in Kigali, Rwanda. 2010.

14. Joint United Nations Programme on HIV/AIDS. Sex workers: Population size estimate: UNdata; 2015 [updated 2024 July 3]. 2015 October 21:[Available from: http://data.un.org/Data.aspx?d=UNAIDS&f=inID%3A111.
15. The World Bank. Population estimates and projections 2020 [cited 2024 July 2]. Available from: https://datacatalog.worldbank.org/dataset/population-estimates-and-projections.
16. Tuyishime E, Kayitesi C, Musengimana G, Malamba S, Moges H, Kankindi I, et al. Population Size Estimation of Men Who Have Sex With Men in Rwanda: Three-Source Capture-Recapture Method. JMIR Public Health and Surveillance. 2023;9:e43114.
17. Twahirwa Rwema JO, Lyons CE, Herbst S, Liestman B, Nyombayire J, Ketende S, et al. HIV infection and engagement in HIV care cascade among men who have sex with men and transgender women in Kigali, Rwanda: a cross-sectional study. Journal of the International AIDS Society. 2020;23:e25604.
18. National Institute of Allergy and Infectious Diseases. Central Africa International epidemiology Databases to Evaluate AIDS: National Institute of Allergy and Infectious Diseases; [cited 2024 July 1]. Available from: https://ca-iedea.org/.
19. Nsanzimana S, Remera E, Kanters S, Forrest JI, Ford N, Condo J, et al. Effect of baseline cd4 cell count at linkage to HIV care and at initiation of antiretroviral therapy on mortality in HIV positive adult patients in Rwanda: A nationwide cohort study. Lancet HIV. 2015;2(9):e376-e84.
20. Nsanzimana S, Mills EJ, Harari O, Mugwaneza P, Karita E, Uwizihiwe JP, et al. Prevalence and incidence of HIV among female sex workers and their clients: modelling the potential effects of intervention in Rwanda. BMJ Glob Health. 2020;5(8):e002300.
21. Ntale RS, Rutayisire G, Mujyarugamba P, Shema E, Greatorex J, Frost SDW, et al. HIV seroprevalence, self-reported STIs and associated risk factors among men who have sex with men: a cross-sectional study in Rwanda, 2015. Sexually transmitted infections. 2019;95(1):71-4.
22. Okal DO, Oyaro B, Zeh C, Desai MA, Samandari T, Chen RT, et al. Effect of point-of-care CD4 cell count results on linkage to care and antiretroviral initiation during a home-based HIV testing campaign: a non-blinded, cluster-randomised trial. Lancet HIV. 2017;4(9):e393-e401.
23. Smith JA, Sharma M, Levin C, Baeten JM, van Rooyen H, Celum C, et al. Cost-effectiveness of community-based strategies to strengthen the continuum of HIV care in rural South Africa: a health economic modelling analysis. Lancet HIV. 2015;2(4):e159-e68.
24. Rwanda Ministry of Health. Rwanda HIV and AIDS national strategic plan 2013–2018: Extension: 2018–2020 2018 [cited 2024 July 3]. Available from: https://rbc.gov.rw/fileadmin/user_upload/stra2019/strategie2019/Rwanda%20Strategic%20Plan%20for%20HIV%20Extended%20to%202020.pdf.
25. Nsanzimana S, Kanters S, Remera E, Forrest JI, Binagwaho A, Condo J, et al. HIV care continuum in Rwanda: A cross-sectional analysis of the national programme. Lancet HIV. 2015;2(5):e208-e15.
26. Rwanda Biomedical Center (RBC). National guidelines for prevention and management of HIV and STIs. Edition 2016 2016 [cited 2024 July 1]. Available from: https://www.rbc.gov.rw/index.php?id=696.
27. Brinkhof MW, Dabis F, Myer L, Bangsberg DR, Boulle A, Nash D, et al. Early loss of HIV-infected patients on potent antiretroviral therapy programmes in lower-income countries. Bull World Health Organ. 2008;86(7):559-67.

28. Nuwagaba-Biribonwoha H, Kiragga AN, Yiannoutsos CT, Musick BS, Wools-Kaloustian KK, Ayaya S, et al. Adolescent pregnancy at antiretroviral therapy (ART) initiation: a critical barrier to retention on ART. J Int AIDS Soc. 2018;21(9):e25178.
29. Haas AD, Zaniewski E, Anderegg N, Ford N, Fox MP, Vinikoor M, et al. Retention and mortality on antiretroviral therapy in sub-Saharan Africa: collaborative analyses of HIV treatment programmes. Journal of the International AIDS Society. 2018;21(2):e25084.
30. Grimsrud A, Cornell M, Schomaker M, Fox MP, Orrell C, Prozesky H, et al. CD4 count at antiretroviral therapy initiation and the risk of loss to follow-up: results from a multicentre cohort study. J Epidemiol Community Health. 2016;70(6):549-55.
31. Johnson LF, Anderegg N, Zaniewski E, Eaton JW, Rebeiro PF, Carriquiry G, et al. Global variations in mortality in adults after initiating antiretroviral treatment: an updated analysis of the International epidemiology Databases to Evaluate AIDS cohort collaboration. AIDS. 2019;33 Suppl 3(Suppl 3):S283-S94.
32. Rwanda Biomedical Center (RBC). Guidelines for HIV prevention, treatment and care in Rwanda. Edition 2022 2022 [cited 2024 July 2]. Available from: https://www.rbc.gov.rw/index.php?id=696.
33. Institut National de la Statistique (INSR) [Rwanda], ORC Macro. Rwanda Demographic and Health Survey 2005 Calverton, Maryland, USA: INSR and ORC Macro; 2006 [cited 2024 July 2]. Available from: http://dhsprogram.com/pubs/pdf/FR183/FR183.pdf.
34. National Institute of Statistics (NISR)[Rwanda], Ministry of Health (MOH)[Rwanda], ICF International. Rwanda Demographic and Health Survey 2010 Calverton, Maryland, USA: NISR/Rwanda, MOH/Rwanda, and ICF International; 2012 [cited 2024 July 2]. Available from: http://dhsprogram.com/pubs/pdf/FR259/FR259.pdf.
35. National Institute of Statistics [Rwanda], Ministry of Finance and Economic Planning [Rwanda], Ministry of Health[Rwanda], ICF International. Rwanda Demographic and Health Survey 2014-15 Kigali, Rwanda: National Institute of Statistics of Rwanda, Ministry of Finance and Economic Planning/Rwanda, Ministry of Health/Rwanda, and ICF International; 2016 [cited 2024 July 2]. Available from: http://dhsprogram.com/pubs/pdf/FR316/FR316.pdf.
36. Rwanda Biomedical Center (RBC). Rwanda population-based HIV impact assessment (RPHIA) 2018–2019: Final Report 2020 [cited 2024 July 1]. Available from: https://phia.icap.columbia.edu/wp-content/uploads/2020/11/RPHIA-Final-Report_Web.pdf.
